# Dust exposure and chronic respiratory symptoms among coffee curing workers in Kilimanjaro: a cross sectional study

**DOI:** 10.1186/1471-2466-11-54

**Published:** 2011-11-24

**Authors:** Gloria Sakwari, Magne Bråtveit, Simon HD Mamuya, Bente E Moen

**Affiliations:** 1 Department of Public Health and Primary Health Care-Occupational and Environmental Medicine, University of Bergen, Kalfarveien 31, 5018 Bergen, Norway; 2 Center for International Health, University of Bergen, Årstadveien 21, N-5020 Bergen, Norway; 3 Department of Occupational and Environmental Health - School of Public Health and Social Sciences, Muhimbili University of Health and Allied Sciences, United Nations Road, Dar es Salaam, Tanzania

## Abstract

**Background:**

Coffee processing causes organic dust exposure which may lead to development of respiratory symptoms. Previous studies have mainly focused on workers involved in roasting coffee in importing countries. This study was carried out to determine total dust exposure and respiratory health of workers in Tanzanian primary coffee-processing factories.

**Methods:**

A cross sectional study was conducted among 79 workers in two coffee factories, and among 73 control workers in a beverage factory. Personal samples of total dust (n = 45 from the coffee factories and n = 19 from the control factory) were collected throughout the working shift from the breathing zone of the workers. A questionnaire with modified questions from the American Thoracic Society questionnaire was used to assess chronic respiratory symptoms. Differences between groups were tested by using independent t-tests and Chi square tests. Poisson Regression Model was used to estimate prevalence ratio, adjusting for age, smoking, presence of previous lung diseases and years worked in dusty factories.

**Results:**

All participants were male. The coffee workers had a mean age of 40 years and were older than the controls (31 years). Personal total dust exposure in the coffee factories were significantly higher than in the control factory (geometric mean (GM) 1.23 mg/m^3^, geometric standard deviation (GSD) (0.8) vs. 0.21(2.4) mg/m^3^). Coffee workers had significantly higher prevalence than controls for cough with sputum (23% vs. 10%; Prevalence ratio (PR); 2.5, 95% CI 1.0 - 5.9) and chest tightness (27% vs. 13%; PR; 2.4, 95% CI 1.1 - 5.2). The prevalence of morning cough, cough with and without sputum for 4 days or more in a week was also higher among coffee workers than among controls. However, these differences were not statistically significant.

**Conclusion:**

Workers exposed to coffee dust reported more respiratory symptoms than did the controls. This might relate to their exposure to coffee dust. Interventions for reduction of dust levels and provision of respiratory protective equipment are recommended.

## Background

Primary coffee processing or coffee curing is done in coffee-growing countries; it involves mechanical cleaning of debris from parchment coffee, hulling, grading green coffee beans (GCB), and packing. Secondary coffee processing involves polishing, roasting, and grinding GCB, and these processes are mainly done in importing countries.

Dust from coffee-processing activities is reported to consist of large and small particles of husks [1], microorganisms and fungi [2], and endotoxin [3]. Several studies have examined dust levels in secondary processing [3-8], but only two studies, from Uganda and Papua New Guinea, have described exposure levels in primary processing [1,9].

Since the 1950s, exposure to coffee dust has been linked to the development of respiratory disorders and allergy [10,11]. In Croatia, a study done among healthy individuals showed that exposure to coffee dust extracts induced a significant decrease in lung function and increased bronchial hyperresponsiveness [12]. Workers in secondary coffee processing (in coffee silos, coffee haulage companies, and coffee manufacturing companies) had reduced lung function and higher prevalence of chronic cough and chronic bronchitis than controls [4,7,8,12]. The few studies [1,9,13] done in developing countries where primary coffee processing (curing) takes place, also indicated that exposed workers have an increased risk of developing airway symptoms. However, these studies have either combined both coffee processing stages (primary processing and roasting) and/or have dealt with workers in green coffee storage warehouses.

Coffee curing factories have been in operation in Tanzania since the 1920s. These factories are still running, and in the last decades new primary processing factories have also been established. There are about 2 million workers employed directly or indirectly in the coffee industry in Tanzania [14]. There are no statistics available on how many of these are directly employed in parchment coffee storage warehouses and in primary coffee factories. With few exceptions [15-18], information on exposure levels and on respiratory symptoms in different industries in Tanzania is not available.

This study aims to assess total dust exposure levels and examine the prevalence of chronic respiratory symptoms among workers in primary coffee factories in the Kilimanjaro region in Tanzania.

## Methods

### Study design and settings

This cross-sectional study was done between November 2008 and January 2009. It involved one relatively new coffee factory established in 1997 (factory A) and an old coffee factory established in the 1920s (factory B). A beverage factory established in 1990 served as a control factory as it was presumed to have no coffee dust and a very low exposure to other types of dust. The coffee factories were selected since they were assumed to represent both new and old technologies that are present in coffee factories in Tanzania. Both coffee factories processed Arabica coffee only. The factories are located close to the Moshi town centre, and the control factory is located 4 kilometres away. Moshi is a small town, and the vehicle traffic is equally low in both of these areas. Thus, ambient air pollution is not considered to have significant impact on respiratory health. All three factories were chosen without any knowledge about the health condition of the workers.

Factory A has both processing machines and storage areas in one room. The machines are relatively new. The factory normally has one working shift from 8:30 a.m. to 5:00 p.m. but in the high season there also is a night shift from 6:30 p.m. to 5:00 a.m. The factory processes approximately 5,500 tonnes of coffee annually. There are 45 workers in the production line and 30 in office work. Activities in this factory include pre-cleaning and destoning, where dust, debris, or unwanted materials are removed mechanically from the parchment coffee. The cleaned parchment coffee is then conveyed to hullers, parchment cover is removed, and husks are blown out to storage tanks while green coffee beans (GCB) are conveyed to graders. Sorting of GCB is done first by using a table grader having six horizontal plates with perforations of decreasing diameters (> 18/64 inches to 8/64 inches) to separate GCB by size to get AA, PB, A, B, C and F grades. The GCB in each grade are then graded by weight using a gravity table to separate into the final grades of heavy and light beans. Both graders have shaking surfaces. The general ventilation system in the factory is natural through three large doors, ventilation openings (0.5 m high) along the walls and along the roof. In addition the sorting table and the gravity table have local exhaust ventilation. Sweeping is done frequently by a dry broom to clean spilled beans.

Factory B is a three-storey building where different processing machines are installed on different floors. Hoppers and parchment storage areas are located on the first and second floors. There is one working shift from 6:30 a.m. to 6:30 p.m. The factory has approximately 60 workers in the production line, including guards and supervisors who have offices in the production area, and 40 office workers including clerks, outside guards, and drivers. The factory processes an average of 5,000 tonnes of parchment coffee annually, which is 10% of its total capacity. The activities in factory B are similar to those in factory A, however with different machine designs. Grading coffee by size is done by using a rotating roller grader which has a cylindrical shape with perforations of increasing diameters (8/64 inches to > 18/64 inches) along the transport flow of the GCB. The catador, which is a pneumatic coffee separator, is used instead of gravity tables to grade the GCB by weight. Husks in the catadors are blown through husk pipes leading to storage tanks. Ventilation in this factory is by means of grids (0.5 m high) all along the outside walls on each floor, and in addition there are three exhaust fans on three walls. Sweeping is done by handheld hoses connected to compressor machines in all processing areas, except in the storage and bulking sections where they use brooms.

In both factories there are workers closely attending the hoppers, hullers, graders, gravity tables and bulking machines. The other workers haul the GCB from the storage area to the machines, and transport the processed GCB from the machines for storage before export. The beverage factory has about 300 permanent employees in production and office, and 100 casual workers. Work in the factory is normally done in two eight-hour shifts, but in the high season there is an additional shift. Ventilation in the factory is natural through large door openings and ventilation openings in the roof. Workers in this factory have permanent tasks depending on the qualification of the worker.

### Study participants

All workers in the production lines of the three factories were eligible participants. There were no female employees in the production lines in any of the factories. The estimated sample size was 160 participants; 80 from the exposed group (factories A and B) and a group of 80 controls. This sample size gave a statistical power of 97% at a significance level of 0.05. The power calculation was based on a study among green coffee workers having a prevalence of chronic cough of 42% vs. 7% among controls [8]. All production workers in factory A (n = 45) were invited to participate. A total of 45 participants were selected randomly from six sections in factory B using personnel lists. The 80 controls were randomly selected from section leaders' lists for the morning shift in that week. The control group lived in the same area as the coffee workers. They were all manual workers strongly indicating that they have similar socioeconomic status.

### Interview for chronic respiratory symptoms assessment

Interview questions used to assess chronic respiratory symptoms were adopted from a standardised questionnaire for assessing respiratory symptoms in adults from the American Thoracic Society (ATS) [19]. Questions used were on whether they usually have cough, wheezing, chest-tightness, breathlessness and chronic bronchitis, whether they ever had past respiratory diseases, smoking habits, and on which type of fuel they used for cooking. These questions were modified to suit the Tanzanian environment. For example, in the set of questions on past respiratory diseases we included tuberculosis as it is prevalent in Tanzania. The modified questions were translated to Kiswahili and then back-translated to English by a different person. The back-translated questionnaire was then compared to the original English version, and corrections were made in the translated questionnaire. The questions about past respiratory disease and respiratory symptoms had a response of "Yes" or "No". Smoking habits were addressed on whether they had ever smoked, when they started smoking, if they are still smoking, number of years they have been smoking, and cigarettes they smoke per day. Participants who had quit smoking within the previous 12 months were considered current smokers. Weight and height were also recorded. Interviews were conducted in Kiswahili with two researchers in factory A, while one of these researchers interviewed all participants from factory B and the controls.

Participants who participated in dust sampling (n = 45) were also asked if they used respiratory protective equipment (RPE) on the sampling day, how frequent they use such equipment, or reasons for not using such protection.

### Dust exposure assessments

Personal "total dust" was sampled throughout the work shift in the breathing zone of participants randomly selected among those who agreed to participate in the questionnaire interview: 22 samples, 23 samples, and 19 samples from factories A, B, and controls respectively. Sampling was done by using Side Kick Casella (SKC) pumps operated at a flow rate of 2 l/minute, attached to electrostatic closed-faced cassettes fitted with 25 millimetre cellulose acetate filters of 0.8 micrometre (μm) pore size. Sampling time ranged from 315 to 720 minutes. The filters were weighted by a Mettler Toledo weighing balance before and after sampling at Eurofins Laboratory in Denmark.

Tanzania does not have its own exposure limit value for organic dust. Hence, we compared values with the recommended eight-hour time-weighted average Norwegian Occupational Exposure Limit (OEL) for total organic dust of 5 mg/m^3 ^[20].

### Ethics

The study was approved by the Regional Committee for Medical Research Ethics, Western Norway, and the National Institute for Medical Research, Tanzania. The management and the workers were informed about the study in meetings. The study methods and aims were also explained to each, individual worker. The workers were free to participate or not. Those who agreed to participate gave written consent.

### Statistical analysis

All data was analysed using Windows Statistical Package for Social Science version 15.0 and 18.0. The significance level was set to p < 0.05. As the exposure data were skewed, they were log transformed before statistical analysis. Continuous variables were compared by using independent t-test. Categorical responses were tested by using Chi-square test; Fisher's exact test was used when any expected number was less than 5. For respiratory symptoms Poisson Regression Model with Robust estimator was used to estimate prevalence ratio (PR) [21] while controlling for confounders; age, smoking, presence of previous lung disease and years worked in dusty factories other than coffee. These confounders were selected based on a significance level of p < 0.2 when comparing coffee workers and controls. Pearson correlations were calculated between age and number of years in the current factory.

## Results

One hundred and fifty two persons agreed to participate; 79 coffee workers and 73 controls. The response rate was 88% for coffee workers and 91% for controls. Two participants from the control group were excluded due to previous exposure to coffee dust.

### Characteristics of the study population

The coffee workers were older compared to controls (t-test p < 0.001) (Table 1). The mean age of the participants correlated with years of work in the current industry (Pearson's correlation coefficient = 0.71, p = 0.01). There were more current smokers among the coffee workers than among controls (Chi-square test p = 0.006). The Body Mass Index was similar in the two groups.

**Table 1:** Characteristics, smoking habits and past respiratory disease for coffee workers and controls

Variable	All coffee workers (n = 79)	Controls (n = 71)
Age (years); AM (range)	40 (19 - 65)	31 (18 - 54) ^a ^**
BMI (kg/m^2^); AM (range)	22.3(17.0 - 30.9)	22.4(17.1 - 30.5)
Weight (kg); AM (range)	65 (51 - 98)	66 (42 - 100)
Height (m); AM (range)	1.70 (1.53 - 1.85)	1.70 (1.54 - 1.91)
Years at present work; AM (range)	12 (0 - 41)	5(0 - 27)
Years worked in other dusty factories; AM (range)	2.8(0 - 43)	1.7 (0 - 17)
**Smoking Habits**		
Never smoked; n (%)	41 (52)	49 (69)
Ever smoked; n (%)	38 (48)	22 (31)
Current smokers; n (%)	23 (29)	10 (14) ^b ^**
Cigarettes smoked per		
day for current smokers; AM (range)	4 (1-15)	3.4 (1-6)
**Previous Respiratory Disease**		
Chest injury; n (%)	1 (1.3)	0
Pneumonia; n (%)	24 (30.4)	13 (18.3)
Pleuritis; n (%)	1 (1.3)	0
Bronchitis; n (%)	1 (1.3)	0
Tuberculosis; n (%)	2 (2.5)	1 (1.4)
Asthma; n (%)	3 (3.8)	6 (8.5)
Other chest problems; n (%)	4 (5)	3 (4.2)
Participants who have had at least one of the respiratory diseases; n (%)	31 (39.2)	21 (29.6)

AM - Arithmetic Mean, ^a ^Independent t test, ^b ^Chi-square test, **p < 0.01, none of the p values was in the range 0.01 < p < 0.05

### Past respiratory diseases

Pneumonia was the past disease with the highest prevalence among both coffee and beverage workers (Table 1). The other types of respiratory diseases were reported less frequently. The prevalence of having had a specific respiratory disease or at least one of these diseases was not different between coffee workers and controls.

### Dust exposure measurements

**Figure 1:**
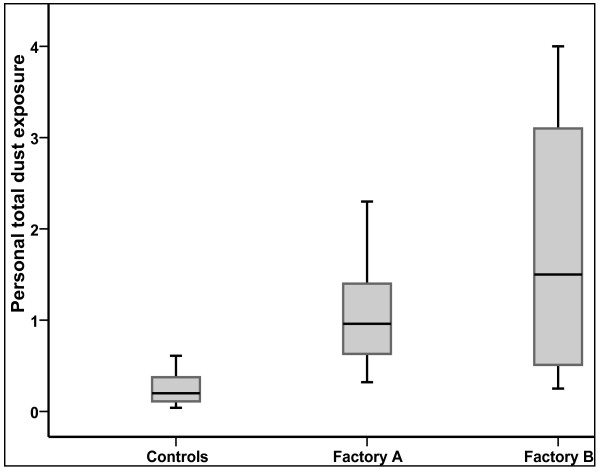
**Personal total dust exposure in the study factories**. Box plot of personal total dust exposure (mg/m^3^) in two coffee factories (A and B) and a beverage factory (control). The boxes include 50% of the measured values and the median values are presented by the lines within the boxes.

Forty four dust samples from coffee factories were analysed. One sample was not analysed as the pump had stopped. Dust exposure in both coffee factories was significantly higher than for controls (independent t-test, factory A to controls, p < 0.001, factory B to controls p < 0.001). Although the variability of dust exposure was higher in factory B than in factory A, dust exposure was not significantly different between these factories (Figure 1). Thus, exposure data from the factories was merged in the following analyses. Personal total dust exposure was GM (GSD) 1.23(0.8) mg/m^3 ^and 0.21(2.4) mg/m^3 ^for coffee factories and controls, respectively (Table 2).

**Table 2:** Personal total dust exposures during different activities among coffee workers and controls

	**Total Dust levels (mg/m**^**3**^**)**				
	
Activity	n	GM (GSD)	AM	Median	Range
All Controls	19	0.21(2.4)	0.29	0.20	0.04 - 1.20
All Coffee workers	44	1.23(0.8)	2.97	1.05	0.25 - 36.0
Sampling and supervision	12	0.68(2.0)	0.86	0.62	0.32 - 2.50
Handling parchment coffee	6	1.02(1.5)	1.07	1.05	0.52 - 1.50
Feeding the hopper	5	1.63(1.7)	1.81	1.60	0.77 - 3.10
Sorting of green coffee	7	1.11(2.4)	1.57	0.95	0.43 - 4.00
Husks handling	2	1.33(1.5)	1.34	1.34	0.97 - 1.70
Sweeping, cleaning coffee grade F	6	8.20(3.7)	14.2	7.55	1.70 - 36.0
Machine repair	6	0.73(2.3)	0.96	0.77	0.25 - 2.40

GM - geometric mean, GSD - geometric standard deviation, AM - arithmetic mean, mg/m^3 ^- milligram per cubic metre

When the dust samples were stratified according to the main task performed during sampling, the highest dust exposures were found in samples taken when cleaning the floor using a broom or compressor and when cleaning coffee grade F (Table 2). However, the coffee workers were not further grouped by task since they rotated daily between different tasks and sections. The mean dust level was lower than the recommended occupational exposure limit in Norway (5 mg/m^3^); however in sweeping and cleaning coffee grade F the mean dust level exceeded this value (Table 2).

### Use of respiratory protective equipment (RPE)

Out of 45 coffee workers, 22 from factory A and 23 from factory B, who were asked about their use of respiratory protective equipment (RPE), a total of 15 (33%) coffee workers reported to use such equipment when working in the dusty area: 7 (32%) in factory A, and 8 (35%) in factory B. The RPE worn was disposable face-piece masks not classified by quality of protection. There were no respiratory protection programs including fit testing in the plants. Two workers in one of the coffee factories had half masks with organic solvent filters. Among the workers who did not have RPE in factory B, 30% used a piece of cloth as protective equipment. Unavailability of the equipment was the reason given by most of the coffee workers (43%) for not wearing the proper RPE. Other reasons reported were; difficulties in breathing through the RPE (30%), feeling safe without the RPE (10%) and that they were used to the dust (17%).

### Prevalence of Chronic symptoms

The coffee workers had a higher prevalence than did controls for all chronic respiratory symptoms (Table 3). Morning cough with sputum and chest tightness was significantly higher among the coffee workers than the controls (23% vs. 10% and 27% vs. 13%, respectively) (Table 3).

**Table 3:** Prevalence of chronic respiratory symptoms among 79 coffee workers and 71 controls

Symptom		Coffee workers n(%)	Controls n(%)	p
Morning cough	All	22(27.8)	14(19.7)	0.26
	Non smokers^a^	15(26.8)	11(18.0)	0.28
Cough day and night	All	38(48.1)	29(40.8)	0.41
	Non smokers	30(53.6)	22(36.1)	0.07
Cough 4-6 days a week	All	24(30.4)	12(16.9)	0.06
	Non smokers	19(33.9)	9(14.8)	0.02
Cough more days in 3 months	All	7(8.9)	3(4.2)	0.33^*b*^
	Non smokers	7(12.5)	1(1.6)	0.03 ^*b*^
Morning cough with sputum	All	18(22.8)	7(9.9)	0.05
	Non smokers	13(23.2)	7(11.5)	0.14
Cough day and night with sputum	All	19(24.1)	11(15.5)	0.22
	Non smokers	17(30.4)	8(13.1)	0.03
Cough 4-6 day a week with sputum	All	12(15.2)	4(5.6)	0.07 ^*b*^
	Non smokers	10(17.9)	2(3.3)	0.01^*b*^
Cough more days in 3 months with sputum	All	6(7.6)	1(1.4)	n.a
	Non smokers	6(10.7)	0	n.a
Dyspnoea I	All	14(17.7)	6(8.5)	0.15
	Non smokers	21(21.4)	5(8.2)	0.06
Dyspnoea II	All	11(13.9)	6(8.5)	0.32
	Non smokers	10(17.9)	5(8.2)	0.17
Wheezing	All	18(22.8)	18(25.4)	0.85
	Non smokers	15(26.8)	11(18.0)	0.28
Chest tightness	All	21(26.6)	9(12.7)	0.04
	Non smokers	20(35.7)	7(11.5)	0.002

^a ^Non smokers comprise 56 coffee workers and 61 controls

Differences between coffee workers and controls are tested by Pearson Chi-square Test

^b ^*Fischer's *exact test. n.a - Not analysed

After adjusting for age, smoking, years worked in other dusty factories than coffee, and experience of any respiratory disease in the past, the prevalence ratio of morning cough with sputum (PR = 2.5, 95% CI 1.0 - 5.9) and chest tightness (PR = 2.4, 1.1 - 5.2) remained significantly higher for the coffee workers compared to the controls. The significance also remained when we adjusted for number of years at the current workplace.

## Discussion

This study shows that total dust exposure among coffee workers is significantly higher than among controls. Coffee workers reported a higher prevalence of morning cough with sputum and chest tightness compared to controls. It is unclear whether this relationship is due to the type of dust (i.e., dust from processing coffee), or the higher total dust levels, or some combination of the two. However, causality cannot be stated due to the cross-sectional design. As a first approach to assess dust exposure in the coffee factories we only measured total dust gravimetrically. Other studies done elsewhere [2,3,7,9,22] have measured either total dust, inhalable dust, or respirable dust, hence we could not directly compare all studies with our results.

Total dust exposure among the coffee workers in our study showed considerable variability ranging from 0.25 mg/m^3 ^to 36 mg/m^3^, with a geometric mean of 1.23 mg/m^3^. The arithmetic mean (AM) level of personal total dust found in the coffee factories in our study, (AM 2.97 mg/m^3^) is lower than the mean personal total dust exposure in green coffee working areas found in a Croatian study (AM 11.2 mg/m^3^), [8] and in an Ugandan study where the range of exposure was 10 - 58 mg/m^3 ^[9]. However, the AM total dust levels found when sorting green coffee beans in our study (1.57 mg/m^3^) is higher than found when handling green coffee in secondary coffee processing in New Orleans (0.44 mg/m^3 ^and 0.48 mg/m^3^) [6]. The dust levels might be expected to vary with a number of factors such as mechanical design of the production areas, ventilation systems and production rates. However, most articles have very little information about the work situation in factories where dust has been measured.

The mean dust levels in this study were lower than the recommended OEL value for organic dust in Norway, which is 5 mg/m^3 ^[20], although in sweeping and cleaning coffee grade F, three individual samples had had higher values (12 mg/m^3^, 30 mg/m^3 ^and 36 mg/m^3^). Despite relatively high exposure to dust in some activities there was little utilisation of respiratory protective equipment. This is similar to what was found in studies done in other dusty factories in developing countries where little or no utilisation of proper respiratory protective equipment was observed [15,23].

In the present study chest tightness and morning cough with sputum were significantly more prevalent among coffee workers than among controls. These symptoms were also found among workers exposed to dust in green coffee silos in a haulage company in Germany, and also in other studies among coffee workers [1,6-9,13]. In New Orleans the mean total dust was lower (0.48 mg/m^3^) than in the present study, but the prevalence of lower respiratory symptoms among exposed coffee workers was still high (32%) [6]. The respiratory symptoms found in our study, as in the other studies, might be associated with dust exposure, as most complaints were from the exposed group. This study and previous studies show that symptoms may be present among exposed coffee workers even at dust levels lower than the recommended occupational exposure limit of 5 mg/m^3 ^[20]. It is difficult to know if the reported symptoms may develop into more serious conditions. Previous studies have shown that work with green coffee may cause both irritation and allergic respiratory symptoms [4,7]: Some studies suggest that coffee workers may develop allergic alveolitis [24] while others suggest asthma [10,22].

Because of their ability to carry out intensive tasks in coffee production, the workers in this study might be considered to be a select group of relatively healthy individuals. Workers who had experienced respiratory discomfort might have changed jobs. Thus, one cannot exclude a possibly healthy worker effect in the coffee factories.

One limitation in our study is the seasonal nature of the coffee crop. The workers are usually at work for eight to nine months a year, depending on the season harvest. Hence, they are free to work elsewhere during the remaining three to four months. Most of the coffee workers work on small-scale family farms during the off-season in coffee processing. However, such farm work is also done by the controls, particularly when they work the night shift at the factory or when they are on holiday.

Self-reporting of symptoms could cause underestimation of symptoms among the workers. Some workers may have feared to lose their jobs if they admitted to have health problems. This was probably reduced in the present study by the private setting of the interview, and the assurance of privacy of the information they provided.

Coffee workers were older and smoked more than the control group. Thus, we adjusted for age and smoking habits when calculating prevalence ratios. In addition, the participants in our study reported a low number of cigarettes smoked per day, indicating a low impact of smoking on reported symptoms. This is similar to what has been found in population studies done in rural and urban settings in Tanzania [25-27]. We did not calculate pack years as the number of cigarettes smoked was low (AM; 4 cigarettes per day). Also among non smokers the prevalence of respiratory symptoms was higher for coffee workers than for controls.

Coffee processing factories in Tanzania have been built between the 1920s and 1990s [28]. Machinery similar to those in the study's factories is presumably found in other coffee factories in this country. Thus, the factories included in this study are probably representative for new and old factories with similar working environments where Arabica coffee is processed.

Interventions for the reduction of dust levels and provision of respiratory protective equipment are recommended, due to the fact that exposed workers suffer from respiratory symptoms. The fact that even relatively low levels were linked to respiratory symptoms might require further investigation on the constituents of the dust from primary coffee factories such as bacteria, fungi, allergens and endotoxins. It is also necessary to study the mechanism behind the development of respiratory symptoms in coffee workers.

## Conclusion

Our results indicate that workers exposed to coffee dust have more respiratory symptoms than do controls. Dust levels in the coffee factories were higher than in the beverage control factory. This is a cross-sectional study; hence, causal relationship should not be concluded. However, the results show that there seems to be a relationship between coffee dust exposure and respiratory symptoms.

## Abbreviations

AM: Arithmetic mean; ATS: American Thoracic Society; CI: confidence interval; GCB: Green Coffee Beans; GM: geometric mean; GSD: geometric standard deviation; OEL: Occupational exposure limit; PR: prevalence ratio; RPE: respiratory protective equipment; SKC pump: Side kick Casella pump.

## Competing interests

The authors declare that they have no competing interests.

## Authors' contributions

GS contributed in data collection, statistical analysis, and the drafting of the manuscript. MB and BEM contributed in the study design, exposure data collection, statistical analysis, and the drafting of the manuscript. SHDM contributed in planning of the project and the drafting the manuscript. All authors gave approval for the manuscript to be published

## Pre-publication history

The pre-publication history for this paper can be accessed here:

http://www.biomedcentral.com/1471-2466/11/54/prepub
